# Myosin binding protein-C slow: a multifaceted family of proteins with a complex expression profile in fast and slow twitch skeletal muscles

**DOI:** 10.3389/fphys.2013.00391

**Published:** 2013-12-25

**Authors:** Maegen A. Ackermann, Aikaterini Kontrogianni-Konstantopoulos

**Affiliations:** Department of Biochemistry and Molecular Biology, School of Medicine, University of MarylandBaltimore, MD, USA

**Keywords:** MYBPC1, skeletal muscle, single fiber expression

## Abstract

Myosin Binding Protein-C slow (sMyBP-C) comprises a complex family of proteins expressed in slow and fast type skeletal muscles. Similar to its fast and cardiac counterparts, sMyBP-C functions to modulate the formation of actomyosin cross-bridges, and to organize and stabilize sarcomeric A- and M-bands. The slow form of MyBP-C was originally classified as a single protein, however several variants encoded by the single *MYBPC1* gene have been recently identified. Alternative splicing of the 5′ and 3′ ends of the *MYBPC1* transcript has led to the differential expression of small unique segments interspersed between common domains. In addition, the NH_2_-terminus of sMyBP-C undergoes complex phosphorylation. Thus, alternative splicing and phosphorylation appear to regulate the functional activities of sMyBP-C. sMyBP-C proteins are not restricted to slow twitch muscles, but they are abundantly expressed in fast twitch muscles, too. Using bioinformatic tools, we herein perform a systematic comparison of the known human and mouse sMyBP-C variants. In addition, using single fiber westerns and antibodies to a common region of all known sMyBP-C variants, we present a detailed and comprehensive characterization of the expression profile of sMyBP-C proteins in the slow twitch soleus and the fast twitch flexor digitorum brevis (FDB) mouse muscles. Our studies demonstrate for the first time that distinct sMyBP-C variants are co-expressed in the same fiber, and that their expression profile differs among fibers. Given the differential expression of sMyBP-C variants in single fibers, it becomes apparent that each variant or combination thereof may play unique roles in the regulation of actomyosin cross-bridges formation and the stabilization of thick filaments.

## Introduction

**Figure 1:**
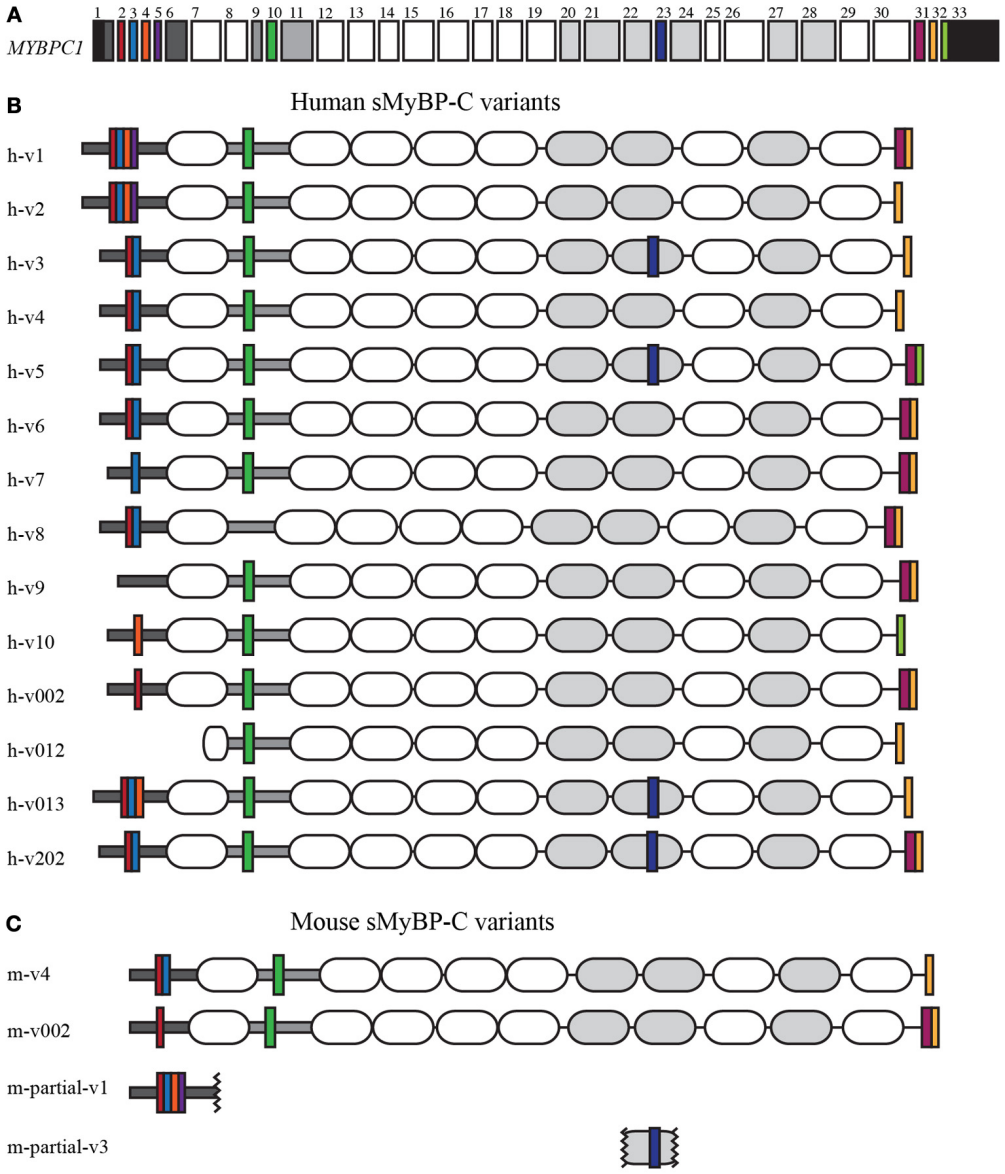
**Human and mouse sMyBP-C variants**. Domain architecture of human and mouse sMyBP-C variants as listed in NCBI, Ensembl, and Vega illustrating their common domains and unique segments. **(A)** Exons 1-33 of the human *MYBPC1* gene; the 5′ and 3′UTRs are denoted in black. Dark gray boxes correspond to exons encoding the Pro/Ala rich region and the M-motif, while the white and light gray boxes represent exons that encode Ig and FNIII domains, respectively. Exons highlighted by a color are alternatively spliced and encode unique regions of the sMyBP-C variants. Alternative splicing of the sMyBP-C transcript in both humans **(B)** and mice **(C)** results in the generation of several variants that share common domains, but also contain unique short sequences in the NH_2_-terminus, the M-motif, the C7 domain, and the COOH-terminus.

Myosin Binding Protein-C (MyBP-C) is a family of accessory proteins that contributes to the assembly and stabilization of thick filaments, and the formation of actomyosin cross-bridges via direct interactions with both filamentous systems (Martyn, 2004; McClellan et al., 2004; De Tombe, 2006; Oakley et al., 2007; James and Robbins, 2011; Ackermann et al., 2013). The slow isoform of MyBP-C, encoded by the single *MYBPC1* gene (Figure 1A), is composed of 7 immunoglobulin (Ig) and 3 fibronectin-III (Fn-III) domains, numbered from the NH_2_-terminus as C1–C10 (Figures 1B,C, shown as white and gray ovals, respectively). The C1 domain is preceded by a sequence of ~50 amino acids that contains a high percentage of proline and alanine residues, referred to as Pro/Ala rich motif (Figures 1B,C, shown as a dark gray horizontal rectangle), and is followed by a conserved linker region of ~100 amino acids, termed M-motif (Figures 1B,C, shown as a light gray horizontal rectangle).

Striated muscles contain three forms of MyBP-C: cardiac, slow skeletal, and fast skeletal, cMyBP-C, sMyBP-C, and fMyBP-C, respectively (Yamamoto and Moos, 1983; Weber et al., 1993; Yasuda et al., 1995; Carrier et al., 1997; Shaffer and Harris, 2009). Single transcripts have been identified for the mammalian cardiac and fast skeletal isoforms, encoding proteins of ~140 and ~130 kDa, respectively (Einheber and Fischman, 1990; Weber et al., 1993; Yasuda et al., 1995). However, sMyBP-C is unique, as there are several mammalian variants that have been reported (Figures 1B,C) ranging in size from ~115 to 130 kDa (Ackermann and Kontrogianni-Konstantopoulos, 2010). These differ by small segments of amino acids within the Pro/Ala rich motif, the M-motif, domain C7, and the extreme COOH-terminus. The different sMyBP-C variants are co-expressed in variable amounts and combinations in both slow and fast twitch skeletal muscles where they co-exist with fMyBP-C (Ackermann and Kontrogianni-Konstantopoulos, 2010, 2011).

MyBP-C localizes along the length of the thick filaments mainly occupying the C-zones of A-bands (Offer et al., 1973; Bennett et al., 1986; Carrier et al., 1997). Similar to its fast and cardiac counterparts, the majority of sMyBP-C variants are targeted to the C-zone. Recent findings, however, have demonstrated that select sMyBPC variants that possess a unique COOH-terminus preferentially localize to the periphery of the M-band where they interact with the giant cytoskeletal protein obscurin and function to stabilize the M-band (Ackermann et al., 2009). Recent studies focusing on sMyBP-C indicated that its NH_2_-terminus (including the Pro/Ala rich motif, domain C1, and the M-motif) interacts directly with actin and heavy meromyosin (HMM), and modulates actomyosin binding and sliding in a variant-specific manner (Ackermann et al., 2013). Interestingly, the Pro/Ala rich motif and the M-motif present in the NH_2_-terminus of sMyBP-C undergo extensive phosphorylation mediated by PKA and PKC (Ackermann and Kontrogianni-Konstantopoulos, 2011); however, the physiological significance of this modification is currently unknown.

Herein we present a comprehensive overview of the known human and mouse sMyBP-C variants, and provide evidence that the expression profile of mouse sMyBP-C differs among fibers of the same skeletal muscle. We also demonstrate for the first time that multiple variants of sMyBP-C are co-expressed within a single fiber, and that their expression correlates with the presence of select myosin isoforms. Lastly, we discuss recent findings indicating the complex regulation of sMyBP-C mediated by alternative splicing and phosphorylation.

## Materials and methods

### Database search

Human and mouse sMyBP-C variants were identified from three prominent databases: NCBI (http://www.ncbi.nlm.nih.gov/), Ensembl (http://www.ensembl.org), and Vega (http://vega.sanger.ac.uk/index.html). To classify the product of a transcript as a complete variant, the transcript should contain at least partial 5′ and/or 3′ UTRs and include defined start and stop codons. Little is known about alternatively splicing within the mouse transcript, therefore any partial mouse sMyBP-C variants that were listed in the above databases were included in the compilation, shown in Figure 1. Most variants, with the exception of human variants 002 and 013 were identified in all three databases, therefore their NCBI accession numbers are noted in Table 1. Human variants 002 and 013 were identified in both Ensembl and Vega databases; the accession number for the Ensembl database is included in Table 1.

**Table 1:** **Human and mouse variants of *sMyBP-C***.

**Variant**	**NH2-terminus**				**M-motif**	**Domain C7**	**COOH-terminus**			**MW (kDa)**	**Protein accession #**
	**Exon 2**	**Exon 3**	**Exon 4**	**Exon 5**	**Exon 10**	**Exon 23**	**Exon 31**	**Exon 32**	**Exon 33**		
h−v1	+	+	+	+	+	−	+	+	3′UTR	131.5	NP_002456
h−v2	+	+	+	+	+	−	−	+	3′UTR	129	NP_996555
h−v3	+	+	−	−	+	+	−	+	3′UTR	128	NP_996556
h−v4	+	+	−	−	+	−	−	+	3′UTR	126.5	NP_996557
h−v5	+	+	−	−	+	+	+	−	+	131.5	NP_001241647
h−v6	+	+	−	−	+	−	+	+	3′UTR	129	NP_001241648
h−v7	−	+	−	−	+	−	+	+	3′UTR	128	NP_001241649
h−v8	+	+	−	−	−	−	+	+	3′UTR	127	NP_001241650
h−v9	−	−	−	−	+	−	+	+	3′UTR	126	NP_001241651
h−v10	−	−	+	−	+	−	−	−	+	128	NP_001241652
h−v002	+	−	−	−	+	−	+	+	3′UTR	127.5	ENSP00000447362^*^
h−v012	−	−	−	−	+	−	−	+	3′UTR	115.5	EAW97667.1
h−v013	+	+	+	−	+	+	−	+	3′UTR	129.5	ENSP00000447660^*^
h−v202	+	+	−	−	+	+	+	+	3′UTR	131	EAW97664.1
m−v4	+	+	−	−	+	−	−	+	3′UTR	126	NP_780627
m−v002	+	−	−	−	+	−	+	+	3′UTR	126.5	NP_00123930 1

* Proteins identified in the Ensembl Database.

### Tissue collection and single fiber isolation

Female C57BL6 mice were euthanized in accordance with protocols approved by the Institutional Animal Care and Use Committee of the University of Maryland School of Medicine and the NIH guidelines, and flexor digitorum brevis (FDB) and soleus muscles were dissected. Primary cultures of FDB and soleus muscle fibers were prepared as previously described (Liu et al., 1997; Calderon et al., 2009), with minor modifications in the preparation of single soleus fibers. In brief, soleus muscles were incubated in DMEM (Gibco, Carlsbad, CA) in the presence of 2 μg/ml type 1 collagenase (Sigma, St. Louis, MO) at 37°/5% CO_2_for 3 h, followed by incubation in DMEM supplemented with 0.75 μg/ml type 1 collagenase at 37°/5% CO_2_ overnight. Treated soleus muscles were gingerly teased apart with fine, fire-polished tweezers and incubated at 37°/5% CO_2_ for an additional hour. Following enzymatic digestion, fresh media was added to the fiber preparations, and single FDB and soleus fibers were picked under an inverted light microscope fitted with a 4x objective. Individual fibers were placed directly into 1x NuPAGE sample buffer (Life Technologies, Carlsbad, CA) containing 10mM NaPO_4_, pH 7.2, 2 mM EDTA, 10 mM NaN_3_, 120 mM NaCl, and 1% NP-40, and analyzed by western blotting.

### Western blotting

Whole muscle FDB and soleus lysates were prepared as previously described, (Ackermann et al., 2009). Briefly, tissues were homogenized in 10 mM NaPO_4_, pH 7.2, 2 mM EDTA, 10 mM NaN_3_, 120 mM NaCl, and 1% NP-40 in the presence of protease inhibitors (Roche Applied Science), incubated on ice for 2 h with occasional mixing, and centrifuged at 14,000 × g for 30 min at 4°C. Approximately 30 μg of protein lysates from each tissue was heated at 90° for 5 min. Similarly, single soleus and FDB fibers were homogenized in the buffer described above, and heated at 90° for 10 min. Both whole muscle and single fiber samples were separated by 4–12% SDS-PAGE using MOPS buffer (Life Technologies). The top part (above ~175 kDa) of each gel was stained with Sypro Ruby total protein stain (Life Technologies) for evaluation of myosin isoforms. The bottom part of each gel (below ~175 kDa) was transferred to nitrocellulose (15V, 16 h, 4°C) and probed with an antibody recognizing epitopes in Ig domain C5, which is common to all known sMyBP-C variants (MYBPC1, Sigma; 1:1000). Each nitrocellulose membrane was stained with Ponceau red dye to evaluate loading. Loading among FDB or soleus fibers was similar with only slight discrepancies, likely due fiber size differences. A total of 48 soleus and 41 FDB fibers were analyzed. The molecular weight of each immune-reactive band was calculated using standard methods as previously described (Anderson et al., 1974).

## Results and discussion

### Alternative splicing of the MYBPC1 transcript results in a complex family of proteins

MyBP-C slow was originally identified as a single protein, similarly to its close relatives, the fast and cardiac isoforms (Weber et al., 1993; Yasuda et al., 1995). Extensive technological advancements of the last decade, however, shed light on the complexity of many transcriptomes revealing the presence of several, previously unidentified, transcript variants originating from single genes. An outstanding example of these efforts is *MYBPC1,* the gene that encodes sMyBP-C. MyBP-C slow is a heterogeneous family of proteins resulting from extensive exon shuffling (Table 1), which primarily takes place in its NH_2_ and COOH termini. Given the great complexity of the sMyBP-C family, we performed a comprehensive analysis of all known sMyBP-C transcripts deposited in the NCBI and Ensembl databases, focusing on the human (h) and mouse (m) variants (v), as they are the most highly studied. For consistency purposes, we use the term “variant” to refer to the different sMyBP-C proteins, while we use the term “isoform” to refer to the cardiac, slow, and fast MyBP-C proteins.

To date, 14 sMyBP-C transcripts have been identified in the human transcriptome, encoding 14 unique variants differing only by short segments of amino acids (Figure 1 and Table 1). Of the 33 exons that make up the human sMyBP-C transcript (Figure 1A), nine of them are shuffled in and out during fifteen splicing events (Figure 1B). In contrast, only two full length and two partial variants have been identified in the mouse transcriptome (Figure 1C). Accordingly, seven splicing events have been identified in the mouse sMyBP-C transcript, resulting in the differential expression of six exons. In humans and mice, the regions that encode the extreme NH_2_ and COOH termini appear to be “hot-spots” for exon shuffling, although domain C7 also undergoes splicing in both species, and the M-motif in humans.

**Figure 2:**
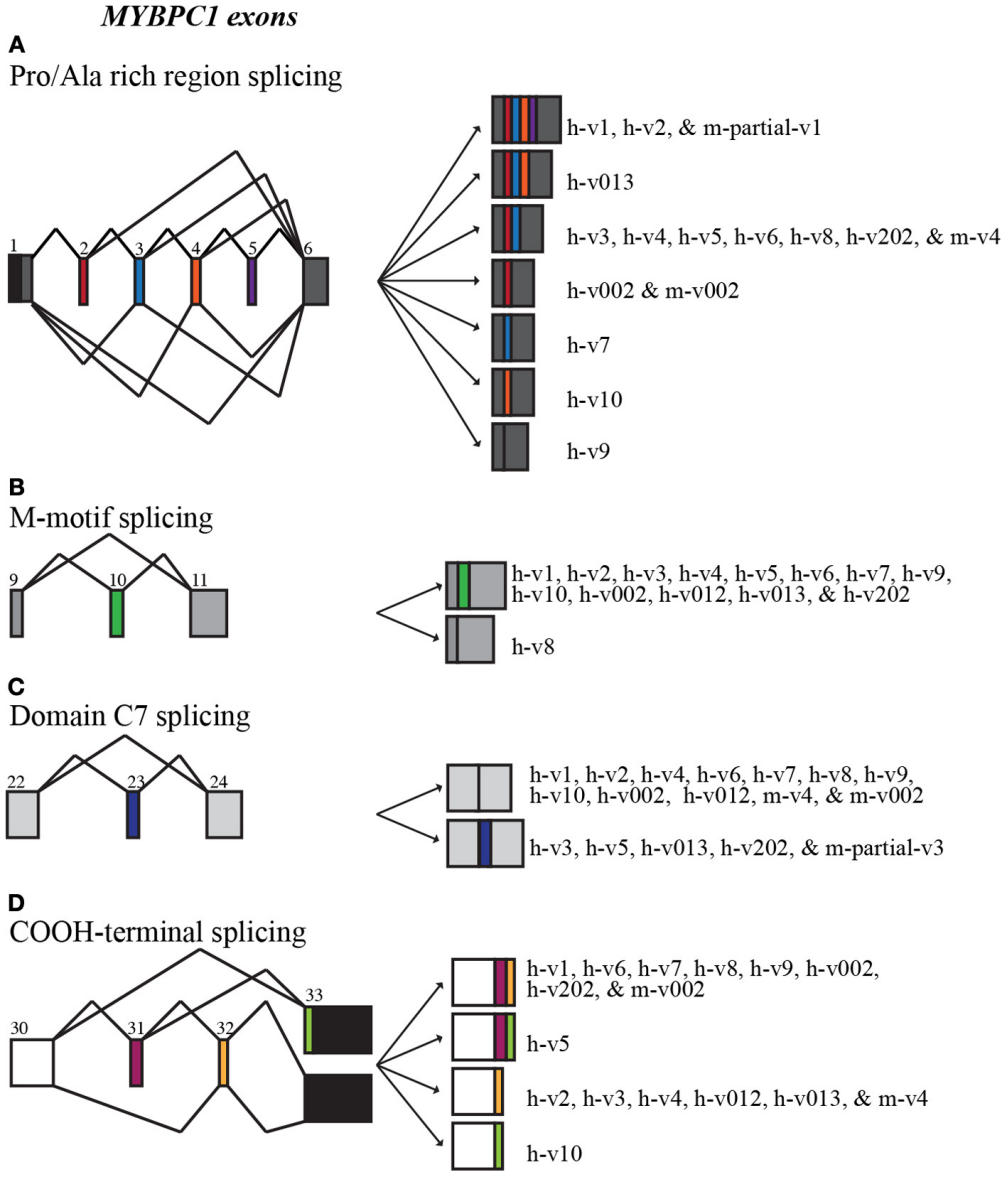
**Alternative splicing scheme of human *MYBPC1***. *MYBPC1* undergoes extensive alternative splicing within the Pro/Ala rich region **(A**; exons 2–5**)**, M-motif **(B**; exon 10**)**, domain C7 **(C**; exon 23**)**, and at the extreme COOH-terminus **(D**; exons 31–33**)**. Exon coloring corresponds to Figure 1.

Splicing within the Pro/Ala rich region at the extreme NH_2_-terminus of human sMyBP-C results in the shuffling of exons 2–5, yielding 7 possible combinations (Figure 2A). Exons 2–5 are arranged in frame and possess 33, 39, 36, and 33 nucleotides, thus encoding 11, 13, 12, and 11 amino acid residues, respectively. sMyBP-C h-v1 and h-v2 contain all four exons, whereas h-v3, h-v4, h-v5, h-v6, h-v8, and h-v202 include exons 2 and 3 but lack exons 4 and 5. Human v013 lacks only exon 5, while h-v002, h-v7, and h-v10 retain only one of the four exons, specifically exon 2, 3, and 4, respectively. Lastly, h-v9 is missing all four exons, while the start codon for h-v012 is in the middle of exon 7, resulting in a protein that lacks the Pro/Ala rich region and the first half of Ig domain C1. Notably, similar splicing events take place in the NH_2_-terminus of the mouse sMyBP-C transcript, giving rise to m-v4 and m-v002, which are homologous to h-v4 and h-v002, respectively. Within the mouse transcriptome, an additional splicing event has been described, leading to the inclusion of all four exons, resulting in the generation of an NH_2_-terminus similar to that of h-v1 and h-v2, however the corresponding full-length mouse variant(s) has yet to be determined.

Additional splicing events occur within the M-motif of the human sMyBP-C transcript (Figure 2B). A simple splicing event in h-v8 results in the exclusion of exon 10 that contains 54 nucleotides, resulting in the generation of an M-motif that is 18 residues shorter than all other known human variants (Figure 2B). To date, this splicing event has not been identified in the mouse sMyBP-C transcript.

Within the region that encodes domain C7, exon 23 consisting of 57 nucleotides is also subjected to alternative shuffling. Variants h-v3, h-v5, h-v013, and h-v202 retain exon 23, and thus contain 19 additional amino acids in the middle of FnIII domain C7 (Figure 2C). The remaining variants lack exon 23 and therefore encode a shorter domain C7. Notably, a similar splicing even has been identified in the mouse sMyBP-C transcript; however, the full length sequence of the relevant variant is still unknown.

The extreme COOH-terminus of human sMyBP-C is another “hot-spot” for complex alternative splicing. In particular, exons 31 and 32 are shuffled in and out resulting in four different combinations (Figure 2D, Table 2). When both exons are retained, exon 31 encodes nineteen amino acids following Ig domain C10, and exon 32 encodes another seven amino acids, a stop codon and the immediate 3′UTR, while exon 33 contributes the remaining 3′UTR. When only exon 31 is included, following the 19 amino acids that it encodes, there are 16 amino acids encoded by exon 33, which also contains a new stop codon and the entire 3′UTR. Conversely, when only exon 32 is retained, it encodes three amino acids following Ig domain C10, a stop codon, and the immediate 3′UTR, while exon 33 provides the remaining 3′UTR. Lastly, when both exons 31 and 32 are spliced out, exon 33 encodes 33 amino acids following Ig domain C10, an alternate stop codon and the entire 3′UTR.

**Table 2:** **Alternative splicing at the extreme COOH-terminus of sMyBP-C**.

**Variants**	**Exon 31**	**Exon 32**	**Exon 33**
h-v1, h-v6, h-v7, h-v8, h-v9, h-v002, h-v202, and m-v002	^*^CDS	^*^CDS/Stop Codon/3′ UTR	3′ UTR
h-v5	^*^CDS	–	^*^CDS/Stop Codon/3′ UTR
h-v2, h-v3, h-v4, h-v012, h-v013, and m-v4	–	^*^CDS/Stop Codon/3′ UTR	3′ UTR
h-v10	–	–	^*^CDS/Stop Codon/3′ UTR

* Coding sequence.

The majority of the human sMyBP-C variants, including h-v1, h-v6, h-v7, h-v8, h-v9, h-v002, and h-v202, retain both exons 31 and 32. On the other hand, h-v5 is the only known sMyBP-C variant that contains exon 31 but lacks exon 32. Moreover, sMyBP-C variants h-v2, h-v3, h-v4, h-v012, and h-v013 lack exon 31, but contain exon 32. Lastly, h-v10 is the only sMyBP-C variant that lacks both exons 31 and 32. Splicing at the COOH-terminus of the mouse sMyBP-C transcript also occurs, however, it has not been as extensively described. Specifically, the COOH-termini of two mouse slow variants, m-v002 and m-v4, match those of h-v002 and h-v4, respectively.

### Distinct combinations of sMyBP-C variants are expressed in single myofibers originating from mouse soleus and FDB skeletal muscles

**Figure 3:**
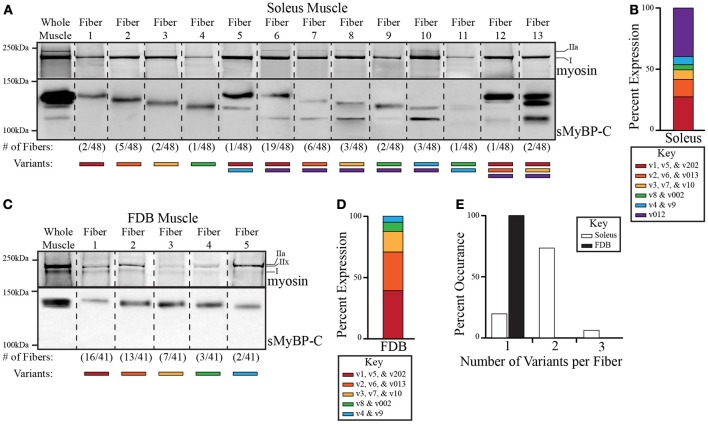
**Analysis of the expression profile of sMyBP-C variants in slow twitch soleus and fast twitch FDB skeletal myofibers**. Endogenous myosin and sMyBP-C proteins were analyzed in single fibers of adult mouse soleus **(A)** and FDB **(C)** muscles. The top part of the gels was stained with SYPRO ruby total protein dye to observe the different myosin isoforms (upper panels), while the bottom part of the gels (lower panels) was processed for immunoblotting using antibodies that recognize epitopes in Ig domain C5, which is present in all known slow variants. Total protein content within each lane was evaluated by Ponceau red staining, and found to be similar. We observed thirteen and five possible expression patterns of sMyBP-C variants in the 48 soleus and 41 FDB fibers examined, respectively. Representative lanes from multiple gels and corresponding blots depicting the expression patterns of myosin and sMyBP-C are shown, and separated by a dotted line; the frequency of each sMyBP-C pattern is noted under the corresponding lane. **(B–D)** Slow variants were grouped according to their predicted molecular weights, and then matched to the appropriate immunoreactive band. The percent (%) expression of each group of slow variants was quantified. **(E)** The number of variants per fiber was calculated and reported as percent (%) occurrence.

We have previously shown that sMyBP-C variants are abundantly expressed in both slow and fast twitch skeletal muscles, and that distinct combinations of sMyBP-C variants are expressed in different skeletal muscles (Ackermann and Kontrogianni-Konstantopoulos, 2010, 2011). An outstanding question, however, is whether single fibers of the same skeletal muscle express one or more sMyBP-C variant(s). To address this question, we examined the expression profile of sMyBP-C variants in 48 and 41 single fibers obtained from mouse slow twitch soleus and fast twitch FDB muscles, respectively. Using western blot analysis and an antibody that recognizes epitopes in Ig domain C5 shared by all known sMyBP-C variants, we found that individual soleus (Figure 3A) and FDB (Figure 3C) fibers contain distinct combinations of sMyBP-C variants. In addition, we quantified their relative abundance (Figures 3B,D), and determined the percentage (%) of fibers expressing one, two, or three immunoreactive bands (Figure 3E). Given that the predicted molecular weights of the different sMyBP-C variants are similar (Table 1), along with the absence of variant-specific antibodies, we were unable to determine the exact composition of sMyBP-C variants in each fiber examined. We therefore calculated the approximate molecular weight of each immunoreactive band according to its electrophoretic mobility, and correlated each band with the respective group of sMyBP-C variants that it may encompass; for instance, soleus fiber 1 contains an immunoreactive band of ~131 kDa, and thus may express v1 (~131.5 kDa), v5 (~131.5 kDa), or v202 (~131 kDa), while FDB fiber 2 contains an immunoreactive band of ~129 kDa, and thus may contain v2 (~129 kDa), v6 (~129 kDa), or v013 (~129.5 kDa). For ease of presentation, we classified the sMyBP-C variants in six groups according to their predicted molecular weights, with group 1 noted in red and including v1, v5, and v202 (131-131.5 kDa), group 2 noted in orange and including v2, v6, and v013 (129–129.5 kDa), group 3 noted in yellow and including v3, v7, and v10 (128 kDa), group 4 noted in green and including v8 and v002 (127–127.5 kDa), group 5 noted in blue and including v4 and v9 (126–126.5 kDa), and group 6 noted in purple and including v012 (115.5 kDa).

Among the 48 soleus fibers that we assayed, we identified 13 different expression patterns of the sMyBP-C variants (Figure 3A). Approximately 74% of soleus fibers expressed variants from two different groups, while ~6% of fibers contained variants from three groups, and the remaining ~20% of fibers expressed variants from one group (Figure 3E, white bars). Of the fibers that contained variants from a single group, group 2 variants (v2, v6, and v013) were most abundantly expressed, being present in ~10% of the total fibers examined (Figure 3A, lane 3). Group 1 (v1, v5, and v202), 3 (v3, v7, and v10) and 4 (v8 and v002) variants were also expressed singly in ~4, 4, and 2% of the total fibers analyzed, respectively (Figure 3A, lanes 2,4,5). Although group 5 variants (v4 and v9) were not observed singly, they were occasionally co-expressed with variants from group 1 (v1, v5, v202), 4 (v8 and v002), or 6 (v012) in ~2, 2, and 6% of the total fibers assayed, respectively (Figure 3A, lanes 6, 11, and 12). Similarly, group 6 variant (v012) was co-expressed with group 1 (v1, v5, and v202), 2 (v2, v6, and v013), 3 (v3, v7, and v10), 4 (v8 and v002), and 5 (v4 and v9) variants in ~40, 12, 6, 4, and 6% of the total fibers examined (Figure 3A, lanes 7–11 and 13,14). Although co-expression of variants from three groups was rare, it did occur among group 1 (v1, v5, and v202), 2 (v2, v6, and v013), and 6 (v012) variants, and group 1 (v1, v5, and v202), 3 (v3, v7, and v10), and 6 (v012) variants in ~2 and 4% of the total fibers tested, respectively (Figure 3A, lanes 13,14). Thus, among the 13 different expression patterns of sMyBP-C that we identified in single soleus fibers, the most prominent one was a combination of group 1 (v1, v5, and v202) and 6 (v012) variants, which accounted for ~40% of the total fibers examined (Figure 3A, lane 7).

Contrary to soleus fibers, all 41 single FDB fibers assayed exhibited the presence of sMyBP-C variants from only one group (Figures 3C,E, black bars), resulting in five different expression patterns. The majority (~71%) of single FDB fibers expressed group 1 (v1, v5, and v202) or group 2 (v2, v6, and v013) variants, which exhibited a ~39 and 32% expression rate, respectively (Figure 3C, lanes 2,3). Group 3 (v3, v7, and v10) and 4 (v8 and v002) variants were also expressed singly with an ~17 and 7% occurrence rate, respectively (Figure 3C, lanes 4,5). Unique to FDB fibers, group 5 variants (v4 and v9) were also expressed solely in ~5% of the total fibers examined (Figure 3C, lane 6), while group 6 variants (v012) were not expressed either alone or in combination with other sMyBP-C group variants. Thus, among the five possible expression patterns of sMyBP-C that we observed in single FDB fibers, group 1 variants were the most common, which accounted for ~39% of the total fibers tested (Figure 3A, lane 2).

Using SYPRO ruby total protein stain, we further correlated the expression profile of myosin isoforms with that of sMyBP-C variants in single soleus and FDB fibers. Soleus muscle predominantly expresses myosin isoforms I and IIa, while FDB muscle mainly contains myosin isoforms IIa, IIx, and I (Calderon et al., 2010; Drzymala-Celichowska et al., 2012). The majority (~82%) of soleus fibers possess both myosin I and IIa isoforms. These are co-expressed with eight out of the thirteen possible combinations of sMyBP-C variants (Figure 3A lanes 3,4, 7–11; Table 3). The remaining 18% of soleus fibers contain solely myosin I, which is co-expressed with the five additional combinations of sMyBP-C variants, (Figure 3A, lanes 2, 5,6, 12–14; Table 3). Conversely, FDB fibers expressing group 1 or group 3 variants (~39 and 17%, respectively) possess myosins I and IIx, fibers expressing group 2 variants (~32%) contain myosins I and IIa, fibers expressing group 4 variants (~7%) encompass myosins I, IIa, and IIx, while fibers expressing group 5 (v4 and v9) variants (~5%) singly contain myosin IIa (Figure 3C, Table 3).

**Table 3:** **Correlation of sMyBP-C variant and myosin isoform expression**.

**sMyBP-C Variant Expression**	**Myosin isoform expression**	
	**Soleus muscle**	**FDB muscle**
v1, v5, and/or v202	I	I and IIx
v2, v6, and/or v013	I	I and IIa
v3, v7, and/or v10	I	I and IIx
v8 and/or v002	I	I, IIa, and IIx
v4 and/or v9	NA	IIa
v1, v5, and/or v202 with v4 and/or v9	I	NA
v1, v5, and/or v202 with v012	I and IIa	NA
v2, v6, and/or v013 with v012	I and IIa	NA
v3, v7, and/or v10 with v012	I and IIa	NA
v8 and/or v002 with v012	I and IIa	NA
v4 and/or v9 with v012	I and IIa	NA
v8 and/or v002 with v4 and/or v9	I	NA
v1, v5, and/or v202 with v2, v6, and	I	NA
v013 and/or with v012		
v1, v5, and/or v202 with v3, v7,	I	NA
and/or v10 and with v012		

### The effect of alternative splicing on the regulation, localization, and function of the sMyBP-C variants

**Figure 4:**
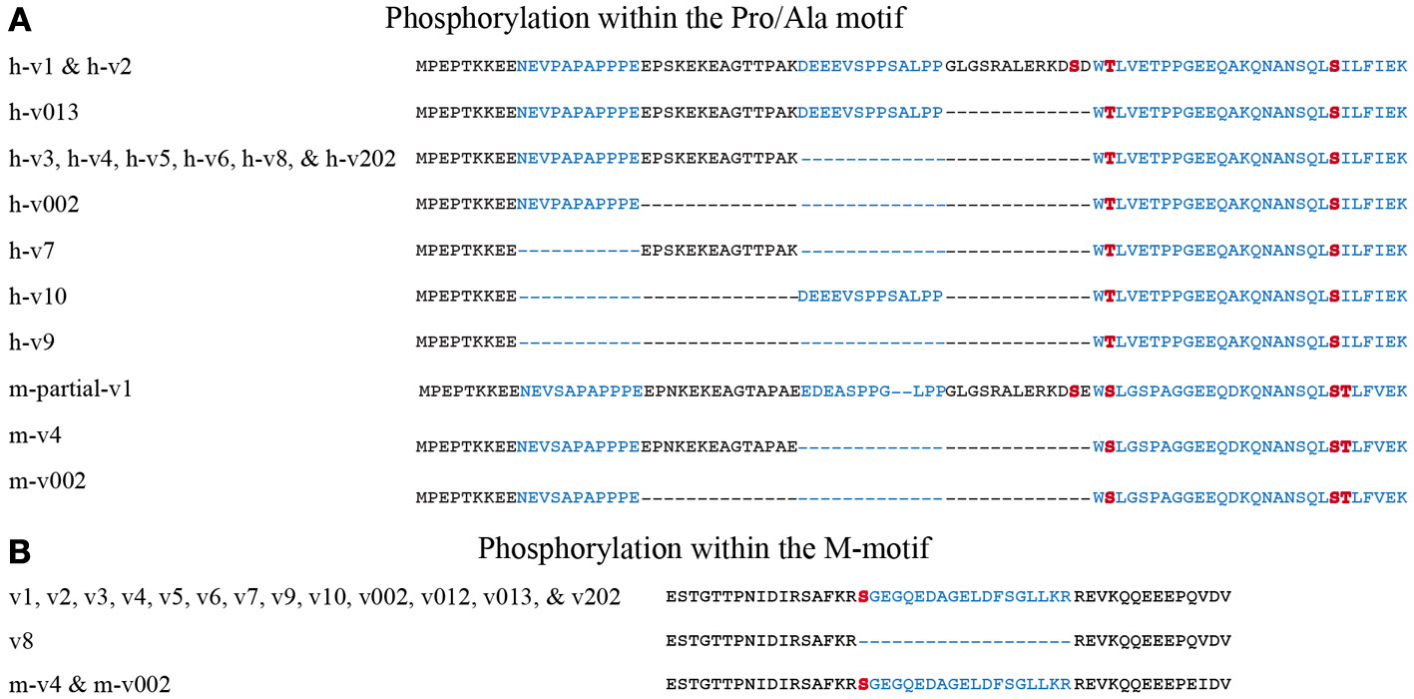
**The NH_2_-terminus of sMyBP-C is subjected to complex phosphorylation**. sMyBP-C is phosphorylated within the Pro/Ala rich region **(A)** and the M-motif **(B)**. Alternating exons are noted in black and blue and phosphorylation sites are shown in red. Amino acid residues h-thr-64/m-ser-62, h-ser-85/m-ser-83, and m-thr-84 are present in constitutively expressed portions of the Pro/Ala rich region. However, h-ser-61/m-ser-59 is located within a segment that is alternatively spliced and therefore unique to h-v1, h-v2, and the NH_2_-terminus of a partially characterized mouse variant (m-partial-v1) matching that of h-v1 and h-v2. Moreover, human h-ser-206/m-ser-204, located in the M-motif is present in all known sMyBP-C variants, with the exception of h-v8 in which the respective exon (exon 10) is skipped.

The sMyBP-C family of proteins is regulated via phosphorylation at its NH_2_-terminus within the Pro/Ala rich region and the M-motif (Ackermann and Kontrogianni-Konstantopoulos, 2011). In particular, within the Pro/Ala rich region of the mouse sequence, residues ser-59 and ser-62 are phosphorylated by PKA and residues ser-83 and thr-84 are phosphorylated by PKC. In addition, ser-204 within the M-motif is a substrate of both PKA and PKC. Four of these five sites are conserved in human sMyBP-C. Specifically, mouse ser-59, ser-83, and ser-204 correspond to human ser-61, ser-85, and ser-206, respectively, while mouse ser-62 aligns with human thr-64 (Figure 4). Mouse thr-84 is not conserved in the human sequence. Although the phosphorylation potential of human sMyBP-C has not been studied, it is likely that it also undergoes phosphorylation at these sites potentially mediated by PKA and PKC (Figure 4).

Alternative splicing of the NH_2_-terminus adds another level of complexity to the phosphorylation status of sMyBP-C. Within the Pro/Ala rich region, all known human (h) and mouse (m) variants carry h-thr-64/m-ser-62 and h-ser-85/m-ser-83 (Figure 4A). However, as these immediately follow the amino acid residues encoded by exons 2–5, which are heavily spliced, their phosphorylation potential is likely dependent on the amino acid sequences preceding them. This suggests that the extensive exon shuffling occurring prior to h-thr-64/m-ser-62 and h-ser-85/m-ser-83 may contribute to the regulation of each slow variant via phosphorylation. Additional experiments are necessary to determine the extent to which alternative splicing affects the targeting of h-thr-64/m-ser-62 and h-ser-85/m-ser-83 by PKA and PKC, respectively.

Contrary to the constitutive expression of h-thr-64/m-ser-62 and h-ser-85/m-ser-83, h-ser-61/m-ser-59, and h-ser-206/m-ser-204 are encoded by exons that are heavily spliced and thus present only in select variants (Figures 4A,B). Human ser-61/m-ser-59, encoded by exon 5 is only present in h-v1, h-v2 and the corresponding mouse variants. The remaining human and mouse variants lack exon 5 and do not express h-ser-61/m-ser-59. Therefore, phosphorylation of h-ser-61/m-ser-59 via PKA in the NH_2_-termini of variants 1 and 2 may contribute to their differential regulation, and distinct modulatory effects on the formation of actomyosin cross-bridges (discussed below). Interestingly, h-v012 starts at exon 7; therefore it lacks all four potential phosphorylation sites present within the Pro/Ala rich region, suggesting that its regulation may differ from that of the other variants. Moreover, h-ser-206/m-ser-204, present in the M-motif, is encoded by exon 10 and is expressed in all variants with the exception of h-v8. Thus, contrary to the remaining sMyBP-C variants, the functional activities of v8 are not regulated via PKA- or PKC-mediated phosphorylation of h-ser-206/m-ser-204.

Early studies have documented that the sarcomeric localization of MyBP-C isoforms is governed by their COOH-termini (Gilbert et al., 1996, 1999; Ackermann et al., 2009). Therefore, small differences in the amino acid composition of the COOH-terminus of sMyBP-C due to alternative splicing may lead to alterations in its subcellular targeting (Table 4). Consistent with this, sMyBP-C variants v2, v3, v4, v012, and v013 that lack exon 31 localize to the C-zone of the A-band (Gilbert et al., 1996, 1999), while sMyBP-C variants v1, v6, v7, v8, v9, v002, and v202 that retain exons 31 and 32 preferentially concentrate to the periphery of the M-band (Ackermann et al., 2009). Moreover, given that the COOH-termini of v5 and v10 are unique, lacking exon 32 and exons 31 and 32, respectively, their subcellular distribution is still unknown.

**Table 4:** **Sarcomeric localization of sMyBP-C variants**.

**Variant**	**A-Band**	**M-Band**	**Unknown**
h-v1		X	
h-v2	X		
h-v3	X		
h-v4	X		
h-v5			X
h-v6		X	
h-v7		X	
h-v8		X	
h-v9		X	
h-v10			X
h-v002		X	
h-v012	X		
h-v013	X		
h-v202		X	
m-v4	X		
m-v002		X	

In light of the effect that alternative splicing has on the phosphorylation potential and subcellular localization of sMyBP-C variants, it is safe to speculate that it also influences their functional activities. Indeed, recent work from our group demonstrated that the ability of sMyBP-C proteins to interact with actin and myosin filaments, and modulate the formation of actomyosin cross-bridges is variant-specific (Ackermann et al., 2013). For instance, sMyBP-C v1 and v2, that retain exons 2–5, exert the most potent regulatory effect on the formation of actomyosin cross-bridges via their NH_2_-terminus by efficiently interacting with both actin and myosin filaments. Moreover, v3, v4, v5, v6, v8, and v202, which retain exons 2–3 also exhibit a strong regulatory effect on the formation of actomyosin cross-bridges by completing with actin filaments for binding to myosin heads. Conversely, v002, which lacks exons 3–5, exerts a moderate regulatory effect by interacting only with HMM. Given that the NH_2_-termini of v013, lacking only exon 5, v7, containing only exon 3, v10, possessing only exon 4, and v9, lacking all exons 2–5, are unique, further studies are necessary to determine their role in the modulation of actomyosin cross-bridges formation.

Our studies have also shown that alternative splicing at the COOH-terminus of sMyBP-C regulates the capacity of sMyBP-C variants to interact with myosin thick and actin thin filaments in a variant-specific manner (Ackermann et al., 2013). Accordingly, sMyBP-C variants v2, v3, v4, v012, and v013 that lack exon 31 interact weakly with the light meromyosin (LMM) portion of myosin and actin thin filaments, while sMyBP-C variants v1, v6, v7, v8, v9, v002, and v202 that retain exons 31 and 32 preferentially interact with LMM. As the COOH-termini of v5 and v10 are unique, lacking exon 32 and exons 31 and 32, respectively, their ability to interact with thick and/or thin filaments is a point of future studies.

## Concluding remarks

Herein we discuss the uniqueness and complexity of the slow isoform of MyBP-C that distinguish it from its lone relatives, the fast and cardiac isoforms. In particular, we focus on the extensive alternative splicing that the *MYBPC1* transcriptome undergoes, leading to the generation of multiple variants that can be differentially phosphorylated. We also present novel evidence indicating that individual soleus and FDB fibers can express more than one slow variant in select combinations, which coincide with the presence of distinct myosin isoforms. Taken together, it becomes apparent that sMyBP-C is a highly complex family of proteins that can be co-expressed in the same muscle and fiber, and potentially sarcomere and thick filament. Dissecting the precise roles that each slow variant plays is an ambitious task that will require a combination of molecular, cellular and biochemical approaches alongside with the generation of the appropriate animal models. The realization of the presence of multiple sMyBP-C variants, which are expressed in distinct combinations among individual myofibers, is the first step toward the elucidation of their differential roles in myofibrillar assembly and actomyosin contractility. Thus, future work is warranted in order to decipher the exact role of each slow variant during development and adulthood in normalcy and disease. Lastly, as a word of caution, given the immense complexity of the slow family of MyBP-C proteins, it is imperative that we tailor our questions, hypotheses, and methodologies in ways that will allow the systematic and comprehensive characterization of the unique properties and regulation of the MyBP-C slow subfamily, without necessarily comparing it to its older relative, the cardiac MyBP-C.

## Author contributions

Concepts in this manuscript were conceptualized by Aikaterini Kontrogianni-Konstantopoulos and Maegen A. Ackermann. The experimental work and drafting the manuscript was performed by Maegen A. Ackermann. Final approval of the manuscript was noted by Aikaterini Kontrogianni-Konstantopoulos.

### Conflict of interest statement

The authors declare that the research was conducted in the absence of any commercial or financial relationships that could be construed as a potential conflict of interest.
